# PSPP: A Protein Structure Prediction Pipeline for Computing Clusters

**DOI:** 10.1371/journal.pone.0006254

**Published:** 2009-07-16

**Authors:** Michael S. Lee, Rajkumar Bondugula, Valmik Desai, Nela Zavaljevski, In-Chul Yeh, Anders Wallqvist, Jaques Reifman

**Affiliations:** 1 Biotechnology HPC Software Applications Institute, Telemedicine and Advanced Technology Research Center, U.S. Army Medical Research and Materiel Command, Fort Detrick, Maryland, United States of America; 2 Computational and Information Sciences Directorate, U.S. Army Research Laboratory, Aberdeen Proving Ground, Maryland, United States of America; 3 Department of Cell Biology and Biochemistry, U.S. Army Medical Research Institute of Infectious Diseases, Fort Detrick, Maryland, United States of America; Tel Aviv University, Israel

## Abstract

**Background:**

Protein structures are critical for understanding the mechanisms of biological systems and, subsequently, for drug and vaccine design. Unfortunately, protein sequence data exceed structural data by a factor of more than 200 to 1. This gap can be partially filled by using computational protein structure prediction. While structure prediction Web servers are a notable option, they often restrict the number of sequence queries and/or provide a limited set of prediction methodologies. Therefore, we present a standalone protein structure prediction software package suitable for high-throughput structural genomic applications that performs all three classes of prediction methodologies: comparative modeling, fold recognition, and *ab initio*. This software can be deployed on a user's own high-performance computing cluster.

**Methodology/Principal Findings:**

The pipeline consists of a Perl core that integrates more than 20 individual software packages and databases, most of which are freely available from other research laboratories. The query protein sequences are first divided into domains either by domain boundary recognition or Bayesian statistics. The structures of the individual domains are then predicted using template-based modeling or *ab initio* modeling. The predicted models are scored with a statistical potential and an all-atom force field. The top-scoring *ab initio* models are annotated by structural comparison against the Structural Classification of Proteins (SCOP) fold database. Furthermore, secondary structure, solvent accessibility, transmembrane helices, and structural disorder are predicted. The results are generated in text, tab-delimited, and hypertext markup language (HTML) formats. So far, the pipeline has been used to study viral and bacterial proteomes.

**Conclusions:**

The standalone pipeline that we introduce here, unlike protein structure prediction Web servers, allows users to devote their own computing assets to process a potentially unlimited number of queries as well as perform resource-intensive *ab initio* structure prediction.

## Introduction

Three-dimensional (3-D) protein structures are critical for the understanding of molecular mechanisms of living systems. Traditionally, X-ray crystallography or nuclear magnetic resonance methods are used to determine the structures of proteins experimentally. In the post-genomic era, where many new complete genomes are available every year and the number of sequences total in the millions, it is impossible to rely on experimental methods alone for structural characterization. Therefore, computational prediction of protein structures is an essential complement. Predicted protein structures help researchers in several ways. First, fold prediction is an important tool for the functional annotation of proteins at the genomic scale [1]–[3]. Moreover, fold and structure predictions can be used to infer binding interfaces [4], potential binding partners [5], and catalytic active sites [6]. In addition, *in silico* drug screening can be performed on close homologues of proteins with known structures [7], [8].

**Figure 1:**
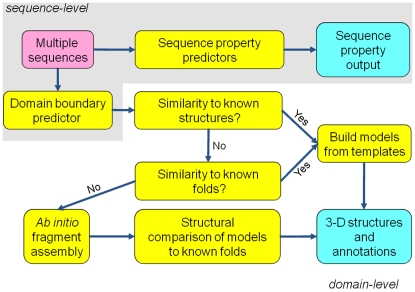
Workflow for the protein structure prediction pipeline given a single query sequence.

The quality of protein structure predictions is directly correlated to the similarity of a query sequence to known protein structures [9]. Procedurally, as shown in Fig. 1, query protein sequences are first divided into manageable chunks. Optimally, domain boundaries are used, but these are often experimentally unknown and must be inferred computationally. Each domain sequence is then compared for similarity against a database of known protein structures, i.e., the Protein Data Bank (PDB) [10], which, to date, consists of over 50,000 entries. If no matches can be detected, fold recognition is instantiated, whereby various characteristics of the domain sequence are predicted, including secondary structure, solvent accessibility, and sequence-specific substitution matrices. These properties are pairwise aligned against the properties of several thousand known protein folds (e.g., the Structural Classification of Protein (SCOP) database [11]). Finally, if no matches are made in this search, the 3-D atomic structure of the protein domain must be built *ab initio*, i.e., the structure must be assembled using energy functions and filters to guide the packing of residues and multi-residue fragments. Because this is a combinatorial process that does not guarantee a globally optimal solution, thousands of models must be proposed. A high-resolution energy function is then applied to ascertain which models might be closest to the native protein [12], [13].

Because computational protein structure prediction is a complex, multi-step process, it requires many diverse tools, often developed by multiple research laboratories, and the expertise to use them. Many Web servers are available for predicting the structure of a given protein sequence [14]–[16]. However, depending on publicly available Web servers is not practical for several reasons. First, they are a shared resource, and one may be limited to a small number of sequence submissions in a given amount of time. Conversely, servers that pre-process entire genomes of protein sequences may be limited to offering only comparative modeling results (e.g., ModBase [17]). Second, the confidentiality of data cannot be guaranteed, i.e., the submitted data and predicted results are often publicly viewable on the server. Third, in some cases, one cannot be assured that the servers are properly maintained and use the most recent databases. Fourth, Web servers that require heavy computational processing to perform *ab initio* fragment assembly (e.g., Robetta [15]) may have query limits or long queues. Finally, servers are often discontinued when the grant that establishes them terminates [18].

Given the sheer amount of genomic sequence data, a standalone pipeline is necessary to process thousands of sequences at a time. Moreover, access to a pipeline's source code allows end users to add or replace components as new techniques, software, or databases become available. Standalone protein structure prediction requires the integration of several tools, which have been generously disseminated by various independent research laboratories. If software is to be distributed over multiple nodes (or cores) in a cluster environment, it is often cost effective to rely on freely available software. While open-source software is desirable, it is not possible in some cases. Fortunately, the x86-based Linux operating system is a common standard among computational laboratories, and pre-compiled binaries tend to perform reliably.

In this work, we introduce a Perl-based software pipeline that integrates multiple free software packages to predict protein structures and structural properties [19]. It is composed of sequence-level and domain-level modules (Fig. 1). Beyond what has been described previously [19], the sequence-level module predicts protein domain boundaries and properties, such as secondary structure, solvent accessibility, transmembrane helices, and structural disorder. The domain-level module produces 3-D atomic protein models and structural annotations via three strategies: homology, fold recognition, and *ab initio* fragment assembly. In addition, multiple sequences can be handled simultaneously via parallelization over numerous processing cores with a message passing interface (MPI)-based job scheduling tool.

## Methods

The pipeline consists of Perl software modules, C-shell scripts, freely available third-party software (albeit many with license agreements), and an in-house implementation of an MPI job scheduler, Pipeman [2]. The main Perl program, *seq_router.pl*, processes command line parameters and calls sequence analysis, domain boundary detection, and domain-processing modules for individual protein sequences. This program can be run on a single processing core or can run on multiple cores on a single computing node using the multithreading capabilities of PSI-BLAST (sequence searching) and PROSPECT II (fold recognition/threading). A second program, *mpi_seq_router.pl*, performs multiple sequence processing in parallel. This program reads a multiple-sequence FASTA file, writes individually labeled sequences into separate FASTA files, and then dispatches individual *seq_router.pl* jobs via Pipeman. Each component is explained in more detail below. Table 1 lists the third-party software and databases that were integrated into this package.

**Table 1:** Third-party and in-house software and databases.

Software or Database	Originating Laboratory	Function	Article	Website
BLAST/PSI-BLAST	NCBI	Sequence search	[20]	[52]
CE	Shindyalov & Bourne	Structural similarity search	[44]	[53]
CHARMM^1^	Karplus	Molecular minimization and scoring	[37]	[54]
DISPrO, SSPrO, and ACCPro	Baldi	Disorder, secondary structure, and solvent accessibility prediction	[27]	[55]
Jackal	Honig	Homology modeler	[30]	[56]
MMTSB	Brooks and Feig	CHARMM front-end/structural analysis	[36]	[57]
MUPRED	Xu	Secondary structure and solvent accessibility prediction	[23]	Bundled
NR/PDBAA	NCBI	Sequence databases	[29]	[58]
PDB	RCSB	Database of biological macromolecular structures	[10]	[59]
PROSPECT II	ORNL	Fold recognition/threading	[34]	[60]
PSIPRED	Jones	Secondary structure prediction	[21]	[61]
Rosetta	Baker	*Ab initio* folder	[41]	[62]
SCOP/ASTRAL	Chothia and Murzin	Database of protein folds	[11]	[63]
SCWRL3	Dunbrack	Side chain placement	[42]	[64]
TMHMM	Viklund	Transmembrane helix prediction	[25]	[65]
DFIRE-AA	In house	Atomic scoring function	[35]	Bundled
FIEFDom	In house	Domain boundary prediction	[28]	Bundled
Pipeman	In house	MPI job distribution tool	[2]	Bundled
PROSPECT II templates	In house	Templates for SCOP 1.73 folds	This work	Bundled
PSPP	In house	Core software for the pipeline	This work	Bundled

1 Optional (requires paid academic or commercial license).

### Protein-level predictions

Before proteins are delineated into separate domains, several properties can be predicted for each query protein, including secondary structure, solvent accessibility, disorder, and the presence of transmembrane helices. Most of these programs require a position-specific substitution matrix (PSSM; a.k.a. “profile”) generated by PSI-BLAST [20] using the *nr* database. For efficiency, we generate the PSI-BLAST profile once and use it for all protein-level predictions. This may slightly degrade the accuracy of certain individual programs, since they are often tuned with specific PSI-BLAST options.

Knowledge of the secondary structure of proteins is helpful in protein classification, understanding protein folding, tertiary structure prediction, and increasing the accuracy of multiple sequence alignments. Although a finer categorization is possible, protein secondary structures are generally classified into three states: helix, strand, and coil. We incorporated three secondary structure prediction tools into the pipeline: PSIPRED [21], SSPro [22], and MUPRED [23].

Solvent accessibility prediction helps in the understanding of protein tertiary structure, antigenic determinants, protein stability analysis, protein structure classification, and protein interaction analysis. We include ACCPro [22] and MUPRED [24] for solvent accessibility prediction in our pipeline. Both programs predict relative solvent accessibility and can be used for classifying residues as exposed or buried using a threshold value.

In addition, transmembrane proteins are an important class of proteins crucial to all multi-cellular organisms. They play a vital role in signal transduction, ion transport, and other significant functions. TMHMM [25] is incorporated in the pipeline to designate different segments of a given protein sequence as intracellular, extracellular, or transmembrane.

Moreover, intrinsically disordered proteins are often responsible for molecular recognition, molecular assembly, protein modification, and entropic chain activities in organisms [26]. In the pipeline, disordered regions in proteins are predicted using DISPro [27]. For each residue, its profile along with the predicted secondary structure (using SSPro) and predicted solvent accessibility (using ACCPro) are input to an artificial neural network that outputs a residue level index from 0 to 9 (where 0 = fully ordered and 9 = fully disordered) [27].

Finally, the query protein sequences are delineated into separate domains using FIEFDom [28], a novel domain prediction method that we have developed. Briefly, FIEFDom performs a PSI-BLAST search of the full protein sequence against a database of known multiple domain structures. A consensus identification of domain boundary regions is accumulated from profile-sequence matches with known structures. If FIEFDom predicts one or more domains longer than 250 residues, which is often a result of failed domain recognition, the user is provided with an option to use Bayesian statistics to break the sequence into smaller blocks.

### Domain-level predictions

After delineation of the query sequence into domains, each domain sequence is routed to homology modeling, fold recognition, and *ab initio* fragment assembly (Fig. 1). If homology modeling is successful, i.e., at least one template is found above a user-specified sequence similarity threshold, the domain module proceeds directly to all-atom scoring. Otherwise, fold recognition is initiated. A fold confidence above a user-specified threshold will trigger model building. After the generation of template-based models by homology and/or fold recognition, all-atom scoring on the models is performed as described below. Finally, if no models are built, *ab initio* fragment assembly (if it has been selected as an option) is instantiated.

In homology modeling, PSI-BLAST [20] is used to find sequences of PDB structures that align to the query sequence. First, the domain sequence is compared against the *nr* database to generate a profile using three iterations of PSI-BLAST. The sequence and profile are then compared against all sequences in the PDB (i.e., the *pdbaa* database [29]), and the top hits are ranked by sequence identity (i.e., the number of exactly matched residues in the alignment divided by the length of the query sequence). Finally, the program Nest, which is part of the Jackal suite of protein modeling programs from the Honig laboratory [30], is used to build homology models from the most promising alignments. Regions of the model that do not align to the template are treated as loops, which must be predicted “*ab initio*.” For this reason, loop regions are often the largest sources of structural errors in homology models. At present, the pipeline only supports one template per comparative model.

The next prediction at the domain level is based on fold recognition. While many good programs exist for this function [31]–[33], few are freely available for download. One of the best options is the free, but closed-source, program PROSPECT II from the Oak Ridge National Laboratory [34]. Fold recognition involves profile-profile alignment of the query sequence to a template database of known folds. In addition to profile, other features, such as secondary structure and solvent accessibility, are evaluated in the alignment procedure. We built PROSPECT II-compatible templates from the SCOP 1.73 database of protein structures [11] (95% sequence similarity filter: *N*∼15,000). The pipeline performs three PROSPECT II passes. In the first pass, a search is performed against all of the SCOP templates. In the second pass, the top-ranked templates (based on a support vector machine-estimated score) are threaded using a more definitive, but costlier, Z-score procedure. In the final pass, templates with the top-ranked Z-scores are threaded using the pairwise interaction option. Finally, Nest is used to build models from the alignments that pass a certain threshold of fold confidence. Fold confidence is computed via an analytical fit to the data points in the table of the original PROSPECT II article [34]. Even with solid confidence scores, 3-D structural models generated by fold recognition will often have quality issues because of slight errors in the template alignment in addition to the loop region problem discussed earlier.

The template-based models resulting from the comparative modeling and fold recognition modules are scored with two procedures. The first scoring program is an in-house implementation of the DFIRE-AA all-atom statistical potential [35]. This potential is derived from an analysis of the inter-atomic distances between pairs of atom types in a large set of known protein structures. The second scoring module first minimizes the model with MMTSB [36] and CHARMM [37] using the PARAM22 [38] all-atom force field and a distance-scaled electrostatic potential with a dielectric constant equal to 4 [39]. The minimized structure is then scored using the PARAM22 force field with the GBMV2 implicit solvent potential [13], [40] and a surface area-based non-polar term. The CHARMM-based scoring module is only available with an academic CHARMM (or commercial CHARMm) license and thus is an optional, albeit valuable, component. The DFIRE-AA and PARAM22/GBMV2 (GB22) scores are output as the raw score divided by the number of residues in the model. This formula is a simple, though imperfect, way to compensate for different-sized models.

If a domain level sequence is too distant from known folds, template-based modeling is no longer a viable option. In this case, the pipeline calls *ab initio* folding, which uses the popular RosettaAbInitio program from the Baker laboratory [41]. The RosettaAbInitio procedure begins by constructing a library of three- and nine-residue fragments from PDB structures with similar sequences, secondary structures, and profiles as stretches of the query sequence. Rosetta assembles these fragments into full-sized protein backbone models using various energy terms and filters. Because the internal united-residue energy function is often unable to discriminate near-native models, the models must be ranked via a post-process. While newer versions of Rosetta offer side chain packing, minimization, and scoring, our pipeline uses its own post-processing algorithm. First, all-atom models are generated by building the side chains onto each backbone model using SCWRL3 [42]. Next, the all-atom models are scored by our in-house implementation of DFIRE-AA [35]. Finally, the top DFIRE-scoring models are minimized and scored using CHARMM, as described above.

Rosetta has been successfully used in remote fold recognition and annotation for genome- scale applications [1], [43]. To classify and annotate the folds of the models that result from the Rosetta code, the structures of the top few models are compared against ASTRAL PDB-style coordinates of the SCOP 1.73 fold library [11] using CE [44]. The top CE matches ranked by Z-score are listed in the output along with the SCOP annotations. If CHARMM is not present on the computer system, the top models as scored by DFIRE are selected instead. The pipeline can parallelize this module over multiple processing cores using the MPI compilation of Rosetta and the Pipeman job distribution tool for the post-processing steps.

### Output formats

As the software evolved, several output options in different formats were developed. The first format is a text-based human-readable output. Hypertext markup language (HTML) output is also available and incorporates a query-template sequence alignment view as well as DFIRE and GB22 scores. Web pages are organized by a hyperlinked directory tree. In addition, tab-delimited output, containing much of the same information as in the HTML output, is generated so that users can import annotation results into spreadsheet applications.

## Results and Discussion

In this section, we demonstrate an application of our pipeline for large-scale protein structure prediction. Then, we show the value of the scoring schemes implemented in our pipeline. Next, we discuss the performance of the pipeline in the CASP7 competition. In addition, we discuss ongoing biological applications using the pipeline. Finally, we discuss computational time and scaling issues.

**Figure 2:**
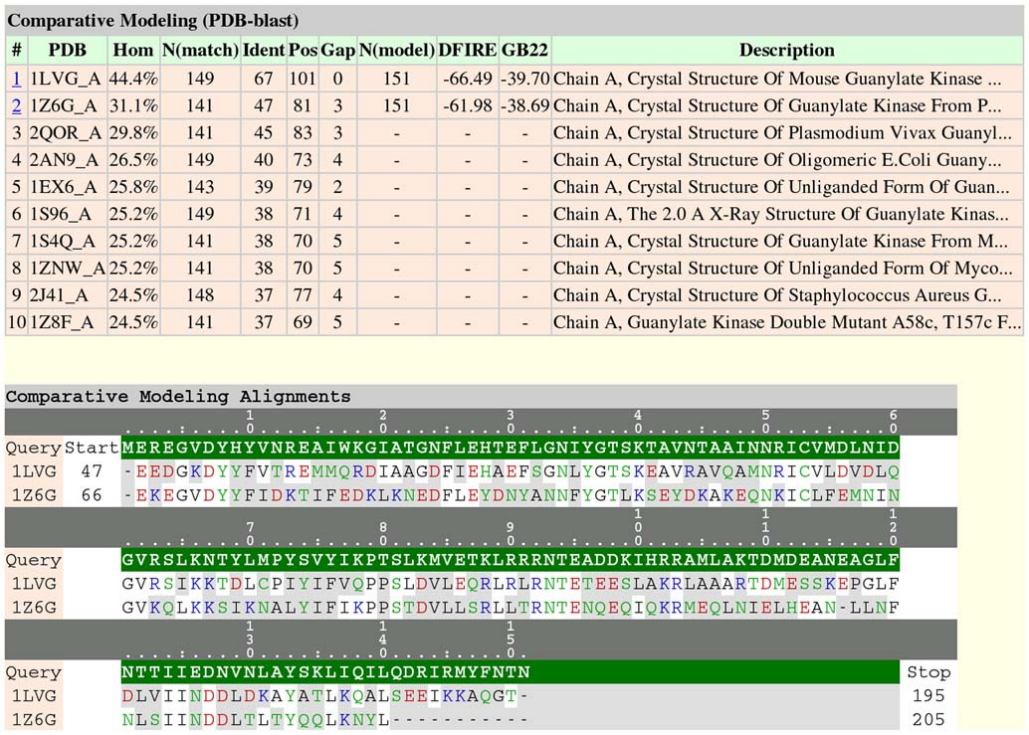
Screenshot of the pipeline-rendered comparative modeling results in hypertext markup language (HTML) format for *variola* protein NP_042212.1. Color-coding for the amino acid letters is as follows: red, acidic; blue, basic; green, polar; and black, apolar.

To demonstrate the use of the pipeline for large-scale processing, we performed structural annotation of the *variola* (smallpox) virus genome [45]–[48], which consists of 197 protein-coding genes. A summary of the results for this run is presented in Table 2. For roughly 10% of the proteins, homology models were produced that might be suitable for drug design (i.e., >50% sequence similarity to a known protein structure). We present the results for three *variola* proteins, which are all labeled “hypothetical,” to show the various outcomes of the prediction workflow (Fig. 1). Figure 2 shows the HTML output for NP_042212.1, one of the *variola* proteins for which a reasonable comparative model could be built. The top model has a 44.4% sequence identity to the known structure of mouse protein guanylate kinase (PDB ID: 1LVG). The GB22 and DFIRE energy scores of the top model were also the highest in rank versus the other comparative models generated. Note that in the HTML output, the index column is hyperlinked to the comparative model in PDB format, if a model is predicted.

**Table 2:** Summary statistics for template-based structure prediction of the proteins encoded by the *variola* (smallpox) genome using the pipeline.

	No. of Proteins	No. of Domains
Total queries	197	355
Sequence similarity to a PDB structure		
>90%	12 (6%)	20 (6%)
Between 50% and 90%	8 (4%)	14 (4%)
Between 30% and 50%	11 (6%)	15 (4%)
Fold recognition (<30% sequence similarity to PDB)		
>90% confidence	32 (16%)	39 (11%)
Between 50% and 90% confidence	21 (11%)	29 (8%)
<50% confidence	113 (57%)	238 (67%)

**Figure 3:**
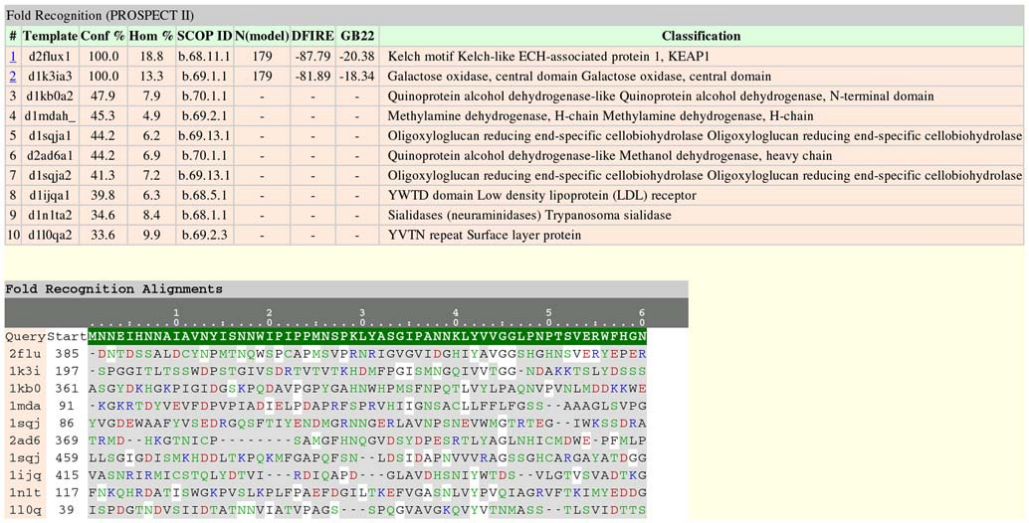
Screenshot of the pipeline-rendered fold recognition results in HTML format for *variola* protein NP_042071.1. This picture truncates the alignment after the first block of 60 residues.

For the protein domains in the *variola* genome that did not have homologous PDB structures, fold recognition was automatically called. An example of a positive fold result is shown in Fig. 3. *Variola* protein NP_042071.1 can be aligned to two SCOP fold templates with confidence scores >50%. Thus, for these two templates, structural models were produced.

**Figure 4:**
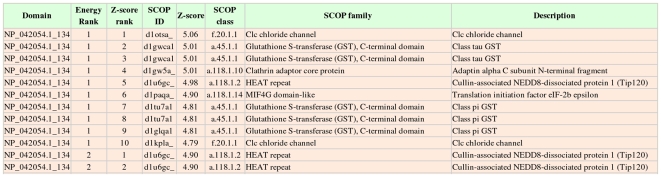
Screenshot of the pipeline-rendered SCOP annotations derived from the *ab initio* results in HTML format for *variola* protein NP_042054.1. The full output (not shown) includes a total of 5 models (ranked by GB22 energy) and the top 10 SCOP matches for each model.

**Figure 5:**
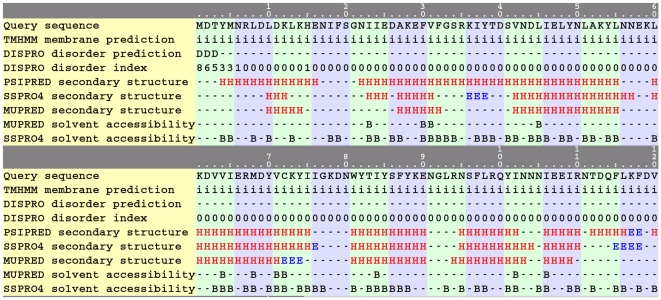
Screenshot of the pipeline-rendered HTML output showing predicted sequence properties for *variola* protein NP_042054.1. i, intracellular; D, disordered; H, helix; E, strand; B, buried.

Perhaps typical of viral genomes, less than half of the proteins encoded by the *variola* genome could be structurally characterized using either homology modeling or fold recognition. Consequently, comprehensive analysis of this genome requires that a large number of the domains be processed via the computationally intensive *ab initio* method. For example, fold recognition on *variola* protein NP_042054.1 did not identify any template with more than 50% confidence. Figure 4 shows the partial HTML output of an *ab initio* run on this protein. *Z*-scores, computed via CE, that are >5 are considered to be matches at the superfamily level [44]. The top-scoring *ab initio* model (energy rank = 1) structurally aligns with a membrane-bound chloride channel and several all-α-helical protein folds. Sequence property predictions for this protein sequence are shown in Fig. 5 (truncated at 120 residues for display purposes). The output includes predictions of transmembrane helices, disorder, secondary structure, and solvent accessibility aligned with the query sequence. Most notably, a membrane-bound fold can tentatively be ruled out because TMHMM did not predict any regions of transmembrane helices.

**Figure 6:**
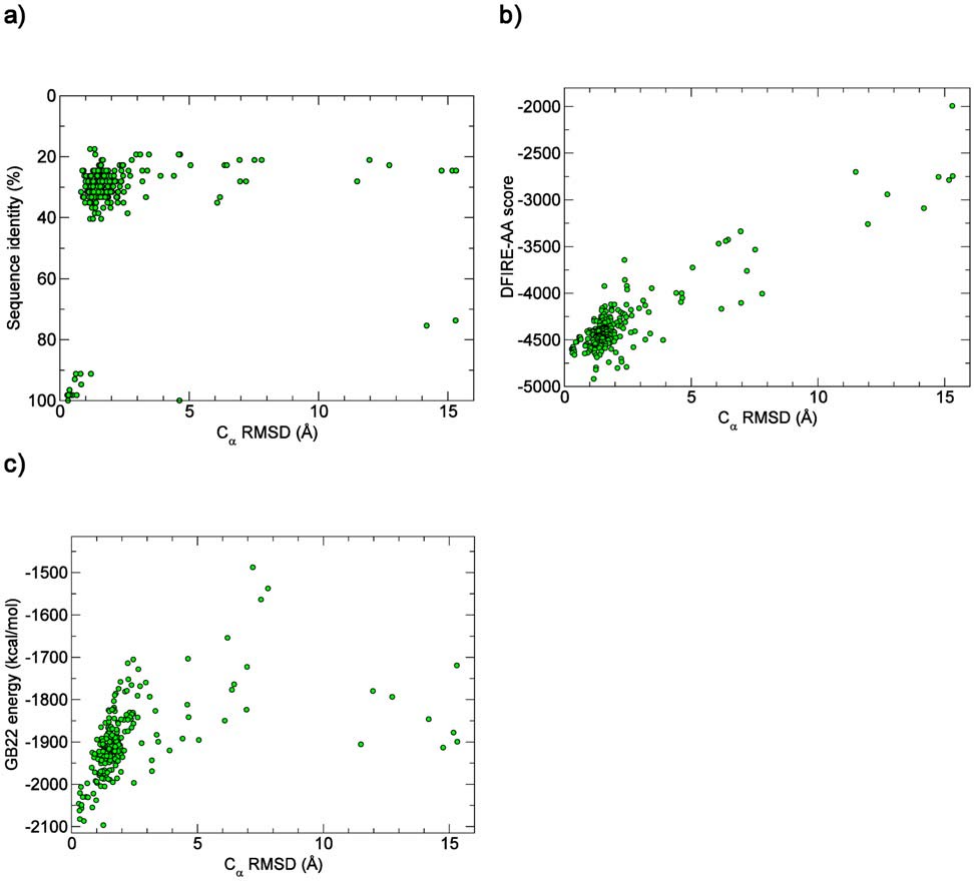
Comparison of various homology model quality criteria against structure accuracy as measured by C_α_, root mean squared deviation (RMSD) for the α-spectrin SH3 domain (PDB ID: 1SHG): sequence identity (a), DFIRE-AA score (b), and GB22 energy (c).

The use of energetic scoring functions, such as DFIRE-AA and GB22, in template-based modeling improves the chances of detecting the most accurate model [49]. As a test case, we predicted 224 all-atom comparative models for the α-spectrin SH3 domain (sequence derived from PDB ID: 1SHG) using only homology modeling. As shown in Fig. 6a, the highest sequence homology hits correctly produced the most accurate Nest-built models. However, suppose that there were no templates with >90% sequence identity. In this thought experiment, percent identity appears to be a poor determinant of model accuracy as measured by the root mean squared deviation (RMSD) of the C_α_-trace between the native X-ray structure and the model (C_α_ RMSD). On the other hand, the DFIRE-AA and GB22 functions (Fig. 6, b and c) show scoring funnels [13] for this query sequence, i.e., as the score improves, so does the model accuracy. Therefore, if only lower sequence identity templates were available, these two scoring functions could aid in selecting the most accurate model.

We participated in the CASP7 experiment in 2006 using an older version of the pipeline and submitted 408 3-D models for 92 targets. Our overall performance ranked in the middle of the 130 participating groups. The noteworthy successes were that one of our homology model predictions and one of our *ab initio* predictions ranked no. 1 in the “Top 1” model category, as measured by the global distance test [50]. We attribute these two cases to the use of the GB22 and DFIRE-AA scoring functions. Our modest performance could be attributed to our lack of advanced loop modeling capabilities or alignment optimization [49] and our reliance on single-template models. Also, at the time, we did not have the domain recognition algorithm FIEFDom to break larger query sequences into more manageable chunks. Regardless, the performance of our standalone pipeline will only improve as new downloadable technologies are shared by research laboratories with the larger community.

Users of the pipeline are currently applying the *ab initio* component to deduce the function of several proteins encoded in virus genomes, including the VP24 protein of Ebola and Marburg viruses [51]. In addition, they are using the pipeline in proteomic surveys of the *Escherichia coli* and *Yersinia pestis* genomes to determine which protein structures can be built by homology, such that protein-protein interactions can be modeled. In addition, the pipeline is helping researchers infer the functions of proteins that, up to now, have been labeled as “hypothetical.”

It is worth discussing the computational effort of the homology modeling and fold recognition run on the *variola* genome. While running in parallel on 64 Xeon 3.0-GHz cores, the pipeline required, on average, nearly 4 CPU-hours per domain when utilizing a shared file system. In contrast, repeating the same calculations using the hard drives of the local nodes instead averaged a much more reasonable ∼1 CPU-hour per domain. We believe that most of the performance degradation on the shared file system can be attributed to PROSPECT II, which uses frequent I/O operations of opening and closing ∼15,000 template and temporary output files for each domain. One solution we are considering is switching to the newly available open-source fold recognition program OpenProspect [38] and modifying it so that it processes large blocks of templates at a time.

In comparison, an *ab initio* run scales well up to 32 processing cores (results not shown). While the Rosetta-MPI component scales almost linearly up to 64 cores, too many simultaneous instances of the structural CE-based similarity search over a shared file system leads to asymptotic limits in speedup. Similar to the situation with PROSPECT II templates, copying the SCOP fold database to the hard drives of the local computing nodes improves parallel performance, albeit with a trade-off of some wall-clock time for copying the database files from shared to local file systems.

### Conclusions

We have introduced a standalone, Perl-based pipeline for protein structure prediction that integrates freely downloadable software components from various academic and government research laboratories. Unlike Web services, which either limit the number of query sequences for processing or perform only a limited subset of prediction techniques, our pipeline allows researchers to harness the power of their own computational resources to perform protein structure predictions at the genomic level. Salient features of our structure prediction software include all-atom scoring, structural annotation of *de novo* models, annotations and sequence alignments in HTML format, and an MPI-parallel framework for large-scale studies.

### Availability and Requirements

- Project name: Protein Structure Prediction Pipeline
- Project download page: http://www.bhsai.org/structure2.html
- Operating system: Linux
- Programming languages: Perl5, tcsh, and C++
